# Supplementary material for: Effect of Nail Grips on Weight Bearing and Limb Function in 30 Dogs 2 Weeks Post Tibial Plateau Leveling Osteotomy
Source: Animals (Basel). 2022 Sep 6;12(18):2312. doi: 10.3390/ani12182312 (PMC9494954; doi:10.3390/ani12182312)
Supplement: Supplementary file 1 [file animals-12-02312-s001.zip › animals-1873407-supplementary.pdf]

**Supplementary Table S1: Client Specific Outcome Measures survey completed on days 1, 7, and 14.**

**Please rate the following: (Compare to before patient's injury)**

1. Walking across slippery floor using the surgical leg

   0= no problem

   1=mildly problematic

   2=moderately problematic

   3=severely problematic

   4= impossible

2. Getting up from resting position using the surgical leg

   0= no problem

   1=mildly problematic

   2=moderately problematic

   3=severely problematic

   4= impossible

3. Consistently using surgical leg during a 5-minute walk using the surgical leg

0= no problem

1=mildly problematic

2=moderately problematic

3=severely problematic

4= impossible
